# NOD mice, susceptible to pancreatic autoimmunity, demonstrate delayed growth of pancreatic cancer

**DOI:** 10.18632/oncotarget.21261

**Published:** 2017-09-24

**Authors:** James Dooley, Emanuela Pasciuto, Vasiliki Lagou, Yulia Lampi, Tom Dresselaers, Uwe Himmelreich, Adrian Liston

**Affiliations:** ^1^ Translational Immunology Laboratory, VIB, Leuven, Belgium; ^2^ Department of Microbiology and Immunology, KU Leuven - University of Leuven, Leuven, Belgium; ^3^ Department of Imaging and Pathology, KU Leuven - University of Leuven, Biomedical MRI/MoSAIC, Leuven, Belgium

**Keywords:** pancreatic cancer, MRI, immune, NOD

## Abstract

Pancreatic cancer is a high mortality form of cancer, with a median survival only six months. There are multiple associated risk factors associated, most importantly type 2 diabetes, obesity, pancreatitis and smoking. The relative rarity of the disease, however, has made it difficult to dissect causative risk factors, especially with related risk factors. A major unanswered question with important therapeutic implications is the effect of immunological responses on pancreatic cancer formation, with data from other cancers suggesting the potential for local immunological responses to either increase cancer development or increase cancer elimination. Due to the rarity and late diagnosis of pancreatic cancer direct epidemiological evidence is lacking, thus necessitating a reliance on animal models. Here we investigated the relationship between pancreatic autoimmunity and cancer by backcrossing the well characterised Ela1-Tag transgenic model of pancreatic cancer onto the pancreatic autoimmune susceptible NOD mouse strain. Through longitudinal magnetic resonance imaging we found that the NOD genetic background delayed the onset of pancreatic tumours and substantially slowed the growth rate of tumours after development. These results suggest that elevated autoimmune surveillance of the pancreas limits tumour formation and growth, identifying pancreatic cancer as a promising target for immune checkpoint blockade therapies that unleash latent autoimmunity.

## INTRODUCTION

Pancreatic cancer is a relatively rare form of cancer, however the high fatality rate makes it the fourth highest cancer in the absolute number of fatalities [1]. The main reason for the high mortality is the late detection of pancreatic cancer, with only 15-20% of cases being diagnosed at a point when they remain resectable, leading to a median survival of less than six months and a five-year survival rate of under 5% [2]. Late detection makes patient study complex, however known risk factors include age, smoking, obesity, lack of physical activity, diet, type 2 diabetes, chronic pancreatitis, cirrhosis and genetic background [3]. What is currently unknown is whether pancreatic autoimmunity is a risk factor or a protective factor in pancreatic cancer development [4].

Cancer and autoimmunity have a complex relationship. Autoimmunity results in increased cell turn-over and the production of proliferative and angiogenic factors during repair. As such, autoimmunity can potentially act as a risk factor for the development of cancers, as indicated by the progression of chronic autoimmune hepatitis to liver cancer [5–7]. Conversely, cancer cells are “self”, giving the anti-self response of autoimmune T cells and B cells the potential to actively eradicate developing cancers [8]. This potential activity of autoimmunity as a protective factor is suggested by the improved rate of melanoma clearance in patients with autoimmune vitiligo, where autoimmune T cells infiltrate into the skin [9, 10]. The relative effect of these two opposite effects needs to be investigated empirically in each cancer setting, with murine models being a more tractable approach than epidemiology for the rarer cancers.

Non-obese diabetic, or NOD, mice develop autoimmune diabetes spontaneously at a high rate when kept in specific pathogen-free conditions. The development of diabetes in NOD mice follows many of the physiological pathways observed in human type 1 diabetics, with the diabetes onset being largely mediated by autoreactive T cells [11]. While NOD mice are classically identified with autoimmunity towards the endocrine tissue of the pancreas, the strain is also intrinsically prone to the development of autoimmunity towards the exocrine tissue, with an increase in the number of circulating autoreactive T cells and heightened traffic through the pancreas, which can be tipped towards pathogenic outcomes through genetic manipulation [12–14]. Here we use the NOD mice, in contrast to the C57BL/6 control strain, to identify the impact of a genetic background prone to pancreatic autoimmunity on the development and growth of pancreatic cancer. Using the Ela1-TAg transgenic model, we find that NOD mice display delayed formation of pancreatic cancer and substantially slower tumour growth rates, suggesting that elevated autoreactivity limits local cancer development and growth.

## RESULTS

**Figure 1:**
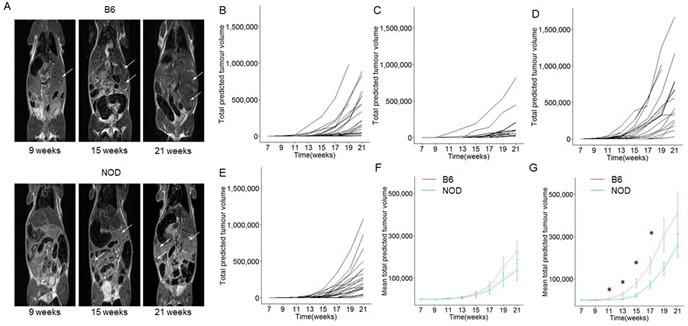
Longitudinal monitoring of pancreatic tumour growth on the B6 and NOD genetic backgrounds B6.TAg^+^ and NOD.TAg^+^ mice were aged and monitored for pancreatic tumour formation and growth by magnetic resonance imaging (MRI). **A**. Represenative MRI scans at 9, 15 and 21 weeks on each strain. Tumours are indicated with an arrow. **B**. Total tumour volumes were estimated for each mouse during the longitudinal observation period. Estimated total predicted tumour volume curves for individual B6.TAg^+^ female mice (*n* = 28), **C**. NOD.TAg^+^ female mice (*n* = 18), **D**. B6.TAg^+^ male mice (*n* = 24) and **E**. NOD.TAg^+^ male mice (*n* = 25). **F**. Aggregate estimated total predicted tumour volume curves for female B6.TAg^+^ and NOD.TAg^+^ mice (*n* = 28, 18). **G**. Aggregate estimated total predicted tumour volume curves for male B6.TAg^+^ and NOD.TAg^+^ mice (*n* = 24,25). * *p* < 0.05.

The Ela1-TAg transgenic strain is a well characterized model of pancreatic cancer development. The mice, developed on the C57BL/6 background, express the T antigen (TAg) under the control of the rat elastase 1 (*Ela1*) promoter, resulting in expression in pancreatic acinar cells. Initial characterization of the C57BL/6 transgenic strain demonstrated pancreatic dysplasia in the embryonic pancreas, with cancer progression after birth to primary pancreatic acinar cell tumor formation as early as 10 weeks of age, with mice moribund around 20-30 weeks of age [15, 16]. In order to determine the impact of genetic susceptibility to pancreatic autoimmunity, we backcrossed the TAg^+^ transgenic strain to the NOD background for more than 14 generations. Pancreatic tumour formation and growth was measured in the parental B6.TAg^+^ strain, resistant to pancreatic autoimmunity, and the newly generated NOD.TAg^+^ strain, susceptible to pancreatic autoimmunity, by longitudinal magnetic resonance imaging (Figure 1A). Tumours were identified in images collected every two weeks, with the total tumour volume estimated from the maximal diameter. Both strains exhibited exponential growth of total tumour volume (Figure 1B-1G), allowing detailed analysis of tumour kinetics. Notably, despite the genetic background predisposed to pancreatic autoimmunity, none of the NOD mice developed autoimmune diabetes (Supplementary Figure 1), due to the conventional housing required for MRI analysis.

**Figure 2:**
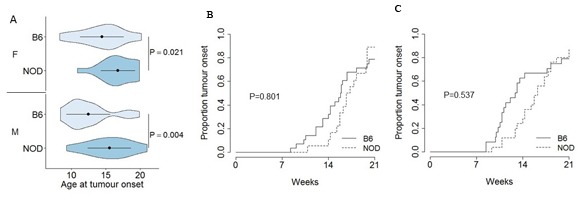
Delayed onset, but normal incidence, of pancreatic cancer on the NOD genetic background B6.TAg^+^ and NOD.TAg^+^ mice were aged and monitored for pancreatic tumour formation and growth by MRI. Age of tumour onset was defined as first detection by MRI. **A**. Violin plots showing the mean, standard deviation and kernel probability density of the age at tumour onset under each condition in male and female B6.TAg^+^ and NOD.TAg^+^ mice (*n* = 24, 28, 25, 18). The *P* values were calculated using two-tailed unpaired *t* test. **B**. Total cumulative incidence of detectable pancreatic tumours in female (*n* = 28, 18) and **C**. male (*n* = 24, 25) B6.TAg^+^ and NOD.TAg^+^ mice. *P* values were calculated using the log-rank test.

Tumour onset was assessed through analysis of the age of first tumour detection, with MRI capable of detecting tumours >3mm in size (data not shown). As male and female B6.TAg^+^ mice showed systematic differences in tumour onset (average of 12 and 14 weeks of age, respectively; Figure 2A), B6.TAg^+^ and NOD.TAg^+^ mice were separated by sex prior to analysis. For mice of both sexes, NOD.TAg^+^ mice shows a systematic delay in tumour development, with first tumour detection delayed by 3-4 weeks (Figure 2A). This delay did not represent a total decrease in tumour incidence, with the penetrance of tumour formation and detection normalizing between the two strains by 18 weeks of age in both female (Figure 2B) and male (Figure 2C) mice. Likewise, histological analysis of tumours did not identify any major differences, including assessment of vasculariation, proliferation and macrophage distribution (Supplementary Figure 2).

**Figure 3:**
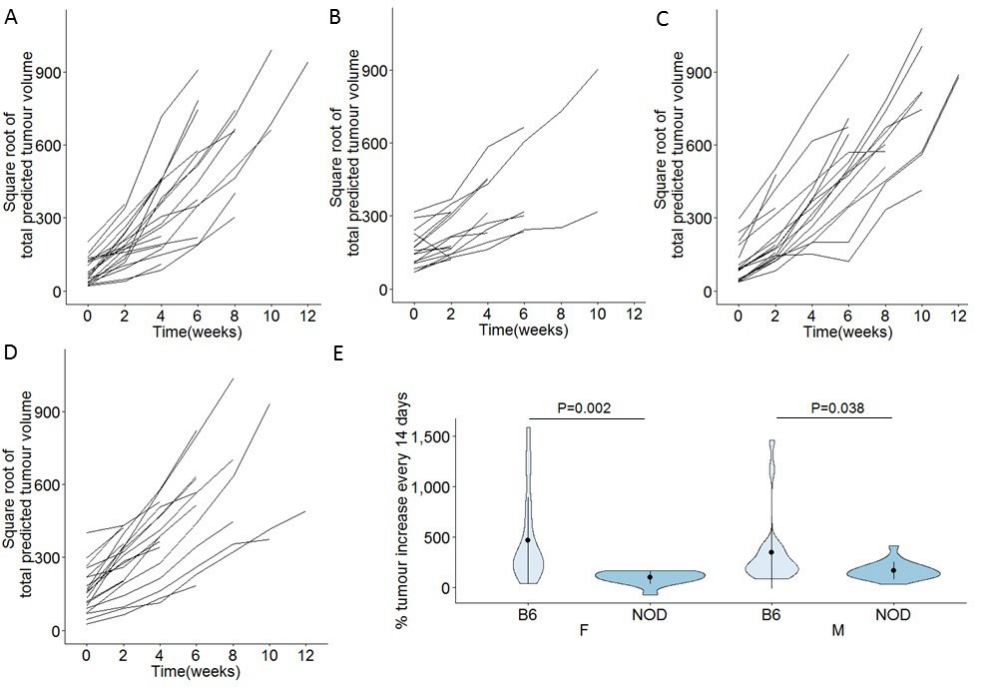
Reduced *in vivo* growth rates of pancreatic cancers on the NOD genetic background B6.TAg^+^ and NOD.TAg^+^ mice were aged and monitored for pancreatic tumour formation and growth by MRI. Total tumour volumes were estimated for each mouse, square root transformed and normalised for time of onset, for **A**. B6.TAg^+^ female mice (*n* = 28), **B**. NOD.TAg^+^ female mice (n = 18), **C**. B6.TAg^+^ male mice (*n* = 24) and **D**. NOD.TAg^+^ male mice (*n* = 25). Time 0 corresponds to the first detected tumour time-point. **E**. Violin plots showing the mean, standard deviation and kernel probability density of the percentage of tumour volume increase every two weeks, average across the observation period, under each condition. *P* values were calculated using two-tailed unpaired *t* test.

**Figure 4:**
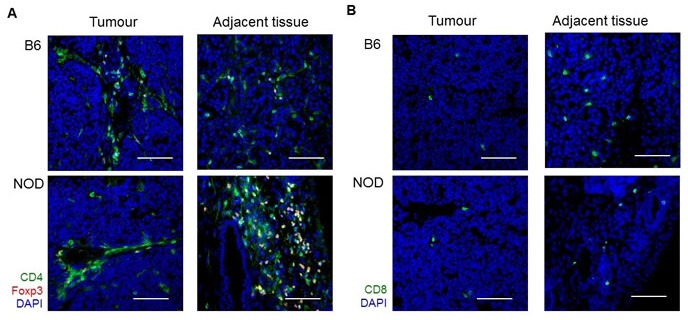
Increased CD4 T cell infiltration adjacent to NOD tumours Tumours from B6.TAg^+^ and NOD.TAg^+^ mice at 12 weeks of age were assessed by histology. Representative images of *n* = 3. **A**. Immunofluorescence for CD4, Foxp3 and DAPI, in the tumour (left) and directly adjacent tissue (right). **B**. Immunofluorescence for CD8 and DAPI, in the tumour (left) and directly adjacent tissue (right). Scale bar = 100μm.

The observed delay in first tumour detection in NOD.TAg^+^ mice, relative to B6.TAg^+^ mice, was suggestive of enhanced pancreatic autoimmunity playing an immunosurveillance role during the early transition from pancreatic dysplasia to tumour formation. These results would also, however, be consistent with reduced tumour growth rates, with slower growth delaying the point at which individuals tumours surpassed the detection threshold. We therefore assessed the growth rate of total tumour volume in each monitored mouse, by normalizing tumour volume to time of first detection and taking into account the exponential growth rates (Figure 3A-3D). Tumour growth was calculated as the percentage increase in total estimated tumour volume every two weeks after tumour detection, averaged across the entire period of observation (Figure 3E). B6.TAg^+^ mice of both sexes averaged 400-500% increase in tumour volume per weeks. NOD.TAg^+^ mice, by contrast, exhibited a marked and significant reduction in tumour growth rates, at 100-200% increase in tumour volume between observation points (Figure 3E). This decrease in the growth rate of the tumours on the NOD background was accompanied by a marked increase in the number of CD4 T cells in the tumour-adjacent pancreatic tissue (Figure 4). Together, these results demonstrate that the NOD genetic background, prone to pancreatic autoimmunity, slows the growth rate of pancreatic tumours in the TAg model.

**Figure 5:**
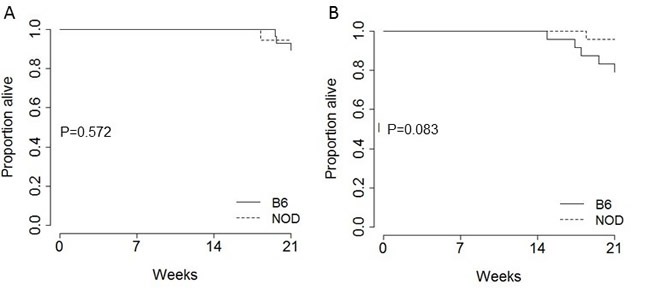
Effect of NOD background on survival in the TAg pancreatic cancer model Kaplan-Meier plots showing the overall pancreatic cancer survival in B6.TAg^+^ and NOD.TAg^+^ mice: **A**. female (*n* = 28, 18) and **B**. male (*n* = 24, 24). *P* values were calculated using the log-rank test.

Finally, the total mortality rate induced by pancreatic tumour was assessed as the age at which excessive morbidity required euthanasia. On the B6 genetic background, tumour-induced mortality was lower in female mice (Figure 5A) than male mice (Figure 5B). Low mortality within the observation period in female mice impeded the ability to determine an effect of genetic background, however in male mice there was a trend towards delayed mortality in NOD.TAg^+^ mice (Figure 4B). While the ethical approval precluded extended observation, such a reduction in early mortality on the NOD genetic background would be consistent with the lower tumour burden observed in these mice on an age-matched basis (Figure 1).

## DISCUSSION

Autoimmunity can have both tumour-promoting and tumour-suppressing impacts on an organ. The net effects of autoimmunity are likely to vary based on the tumour type, the degree of susceptibility to T cell targeting and the reliance of the tumour on oncogenic environmental stimuli that can be provided by inflammation. While the data on the net effect of autoimmunity on each tumour type is still lacking, it is at least notable that the tumour type with the best evidence for suppression by autoimmunity, melanoma, is also among the most responsive to immunotherapy [17]. In the case of pancreatic cancer, the evidence of association with autoimmunity is scant and ambiguous. There is an association of pancreatic cancer with diabetes and chronic pancreatitis [3], but in both cases autoimmunity is the rarer form of the disease, and there is insufficient numbers to identify a correlation, positive or negative, between pancreatic cancer and either autoimmune diabetes or autoimmune pancreatitis [4]. Furthermore, the late detection of pancreatic cancer confounds the direction of causality even when a clear correlation is observed – for example it has been convincingly argued that at least a fraction of the association is a reverse causality, with undiagnosed pancreatic cancer driving diabetes and pancreatitis [18].

At face value, the reduced tumour growth rate in NOD.TAg^+^ mice suggests that the increased autoimmune potential in NOD mice curtails the growth of pancreatic cancer, with anti-tumour immunity being the most direct potential mechanism. The active burden of autoreactive T cells present in the pancreas cannot be directly measured, until a full catalog of relevant pancreatic autoantigens allows tetramer assessment, however the NOD strain has been characterized to have an elevated number of autoreactive T cells circulating through the pancreas, reactive against both endocrine [11] and exocrine [12–14] self-antigens. Here we found a large number of CD4 T cells in the pancreatic tissue adjacent to the healthy tissue, a “guilt by association”, although the antigen-specificity of these cells remains unknown. However it should be noted that NOD and B6 mice are highly divergent strains, with profound genetic differences independent of the immune system. In the pancreas, NOD mice have been shown to have altered development [19], beta cells which exhibit increased fragility [20] and depressed neurogenic inflammation induction by pancreatic sensory neurons [21]. It is therefore a distinct possibility, albeit a less parsimonious one, that the reduced growth rate is caused by intrinsic differences in the nascent tumour cell on the NOD background rather than by the enhanced autoimmune presence.

Additional studies will be needed confirm our finding that autoimmune potential is associated with slower pancreatic cancer growth, ideally using alternative models of both autoimmunity and pancreatic cancer. Confirmation of such a result would, however, provide a profound insight not only into the biological processes, which initiate and regulate pancreatic cancer development and growth, but also for therapeutic potential. Classical cancer treatment approaches, namely surgery, chemotherapy and radiotherapy, have proven largely ineffective in pancreatic cancer, with survival rates remaining essentially unchanged in recent decades [1]. This has led to increased interest in the potential of harnessing the host immune response to control pancreatic cancer [22]. Certainly higher numbers of T cells present in the tumours of pancreatic ductal carcinomas correlates with superior survival [23], and pancreatic cancer patients possess circulating anti-tumour T cells [24], supporting the promise of cancer immunotherapy. However to date attempts to increase endogenous anti-tumour immunity in pancreatic cancer patients has shown little sign of therapeutic success [22]. Our results, indicating the potential for pancreatic autoimmunity to control tumour growth, suggest that the immune checkpoint inhibitor trials currently underway may exhibit more success as these strategies essentially serve to unleash the latent autoimmunity encoded in the immune system [25]. One note of caution, however, is warranted in the observation that even on the autoimmune-prone genetic background, cancer growth was restrained but spontaneous remission was not observed. It is therefore likely that immune checkpoint inhibition will require strategic combination therapy in order to clear established tumours in patients. Supporting this hypothesis, a recent study using transplantable pancreatic adenocarcinomas in mice has found an enhanced immune response when combining radiotherapy with PD-L1 blockade [26], which unleashes latent immunity.

## MATERIALS AND METHODS

### Mice

Ela1-TAg mice, with transgenic expression of the SV40 large T Antigen under the Elastase-1 mouse acinar cell promoter, were purchased from Jackson on the C57BL/6 background [15, 16] (B6.TAg^+^). Ela1-TAg mice were backcrossed to the NOD background (sourced from Taconic) for more than 14 generations (NOD.TAg^+^). Mice are both strains were bred in specific pathogen-free conditions and housed in conventional conditions. Mouse-weight and blood glucose were monitored throughout the experimental process. Mice were used in accordance with the University of Leuven Animal Ethics Committee.

### Serum analysis

Serum samples were collected by retro-orbital bleed and were stored at −80°C. Insulin concentrations in serum were measured by ELISA (cat. no. 10-1247-01; mouse insulin ELISA, Mercodia) according to the manufacturer's instructions.

### Imaging

TAg^+^ mice were scanned with magnetic resonance imaging (MRI) every two weeks from 7 weeks of age. Mice were anesthetized using isoflurane during scan time, with temperature and respiration maintained at 37°C and > 40 min^−1^, respectively. Images were acquired using a Bruker Biospin 9.4 Tesla Biospec small animal MR scanner (Bruker Biospin, Ettlingen, Germany) equipped with an actively shielded gradient set of 600mT/m, using a respiration triggered spin echo sequence (RARE) with 50 continuous slices of 0.5 mm thickness in interlaced mode (acquisition parameters: repetition time = 6000 ms, echo time = 15.9 ms, field of view = 4.0 × 6.0 cm, a matrix of 200 × 400, two dummy scans and two averages). For radio-frequency irradiation and detection, a 7.2 cm quadrature resonator (Bruker Biospin, Ettlingen) was used.

### Histology

Histology fresh frozen sections were fixed in 4% PFA and stained followed by hematoxylin and eosin staining. For immunofluorescence, pancreatic tumours were fresh frozen in OCT, fixed in 4% PFA or acetone, and stained according to manufacturer's protocol. Sections were stained using the polyclonal Ki67 (Rabbit Anti-Ki67, Abcam ab15580), Foxp3 (1054C, R&D ab15580), Trypsin 3 / PRSS3 (Goat anti-Trypsin, R&D Systems MAB8214), CD4 (GK1.5, in-house hybridoma supernatant), CD8 (5H10-1, BioLegend 100802), F4/80 (in-house hybridoma supernatant) and MECA-32 (Rat anti-PLVAP, in-house hybridoma supernatant). For immunofluorescence the following detection antibody were used: Donkey anti-Rabbit 488 (Molecular Probes), Donkey anti-Goat Alexa Fluor 546 (Life Technologies), Donkey anti-Rat 488 (Life Technologies) and DAPI (Life Technologies). Images were acquired using a Ziess LSM 780 confocal microscope.

### Data and statistical analysis

Tumour onset was defined as the age of first tumour detection by MRI. Death was defined at the age at which the mouse was euthanized due to excessive morbidity (weight loss >20%, mobility impairment, degeneration of behavior or general condition). MRI analysis was performed using ImageJ (National Institute of Health, Bethesda, MD, USA). For each tumor, the mean area was calculated at the maximum radius and a tumor volume prediction was made using the formula: 4/3*area*√(area/π). Statistical analysis was made with R (https://www.r-project.org/version 3.1.2). Cumulative incidence curves were generated using the R package “survplot” with the fun=function(x) {1 - x} argument [27]. Survival curves were generated using the Kaplan-Meier method in the R “survplot” package. Comparison of cumulative incidence and survival distribution between two samples was performed using log-rank test implemented in the R “survdiff” package [28].

## SUPPLEMENTARY MATERIALS FIGURES


