# Supplementary material for: NOD mice, susceptible to pancreatic autoimmunity, demonstrate delayed growth of pancreatic cancer
Source: Oncotarget. 2017 Sep 24;8(46):80167–74. doi: 10.18632/oncotarget.21261 (PMC5655187; doi:10.18632/oncotarget.21261)
Supplement: Supplementary file 1 [file oncotarget-08-80167-s001.pdf]

NOD mice, susceptible to pancreatic autoimmunity, demonstrate delayed growth of pancreatic cancer

SUPPLEMENTARY MATERIALS

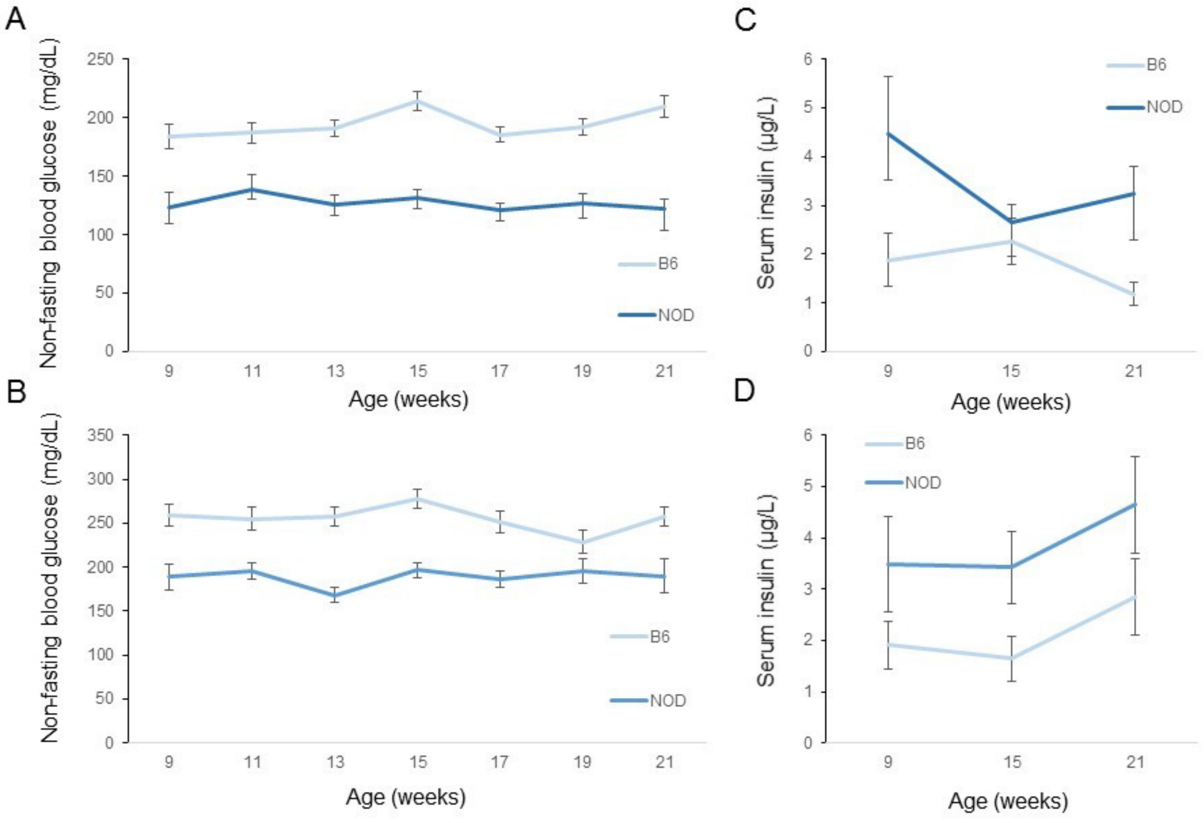

Supplementary Figure 1: Longitudinally stable blood glucose and insulin homeostasis in pancreatic acinar carcinoma mice. B6.TAg+ and NOD.TAg+ mice were aged and monitored for non-fasting blood glucose and serum insulin levels. (**A**) Non-fasting blood glucose levels in B6.TAg+ and NOD.TAg+ female mice (n=28, 18) and (**B**) male mice (n=24, 25). (**C**) Non-fasting serum insulin levels in B6.TAg+ and NOD.TAg+ female mice (n=9, 10) and (**D**) male mice (n=8, 8).

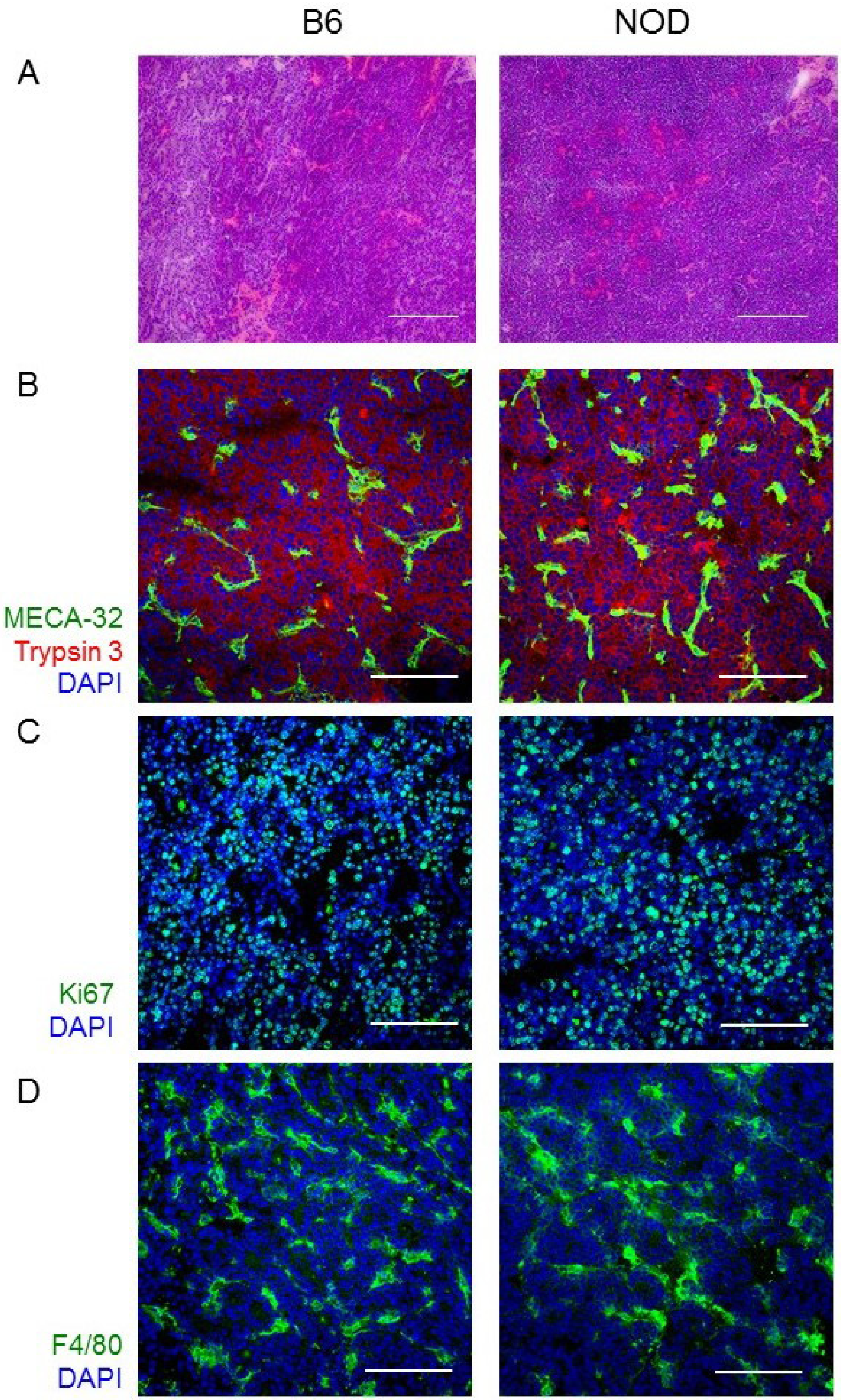

Supplementary Figure 2: Unaltered structural components of tumours in B6 and NOD mice. Tumours from B6.TAg+ and NOD.TAg+ mice at 12 weeks of age were assessed by histology. Representative images of n=3. (**A**) Haematoxylin and eosin staining. Scale bar = 250μm. (**B**) Immunofluorescence for MECA-32, Trypsin 3 and DAPI; (**C**) Ki67 and DAPI; (**D**) F4/80 and DAPI. Scale bar = 100μm.
